# Importance of Bridging Molecular and Process Modeling to Design Optimal Adsorbents for Large-Scale CO_2_ Capture

**DOI:** 10.1021/acs.accounts.3c00478

**Published:** 2023-12-29

**Authors:** Lourdes F. Vega, Daniel Bahamon

**Affiliations:** Research and Innovation Center on CO_2_ and Hydrogen (RICH) and Department of Chemical and Petroleum Engineering, Khalifa University, P.O. Box 127788, Abu Dhabi, United Arab Emirates

## Abstract

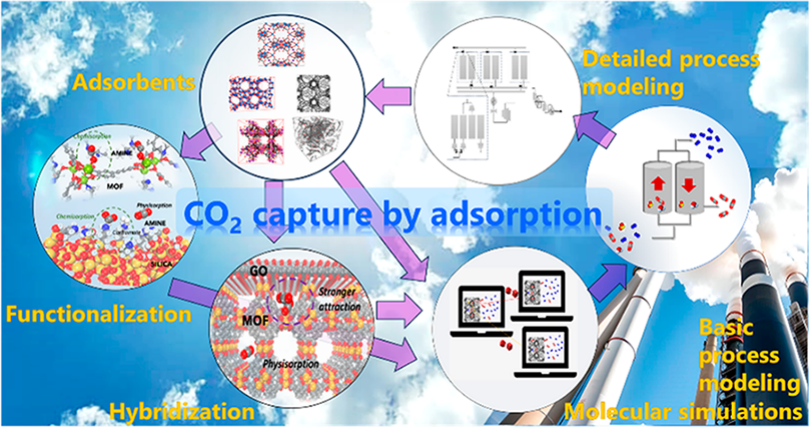

Carbon capture, utilization, and storage have been identified as key technologies to decarbonize the energy and industrial sectors. Although postcombustion CO_2_ capture by absorption in aqueous amines is a mature technology, the required high regeneration energy, losses due to degradation and evaporation, and corrosion carry a high economic cost, precluding this technology to be used today at the scale required to mitigate climate change. Solid adsorbent-based systems with high CO_2_ capacities, high selectivity, and lower regeneration energy are becoming an attractive alternative for this purpose. Conscious of this opportunity, the search for optimal adsorbents for the capture of CO_2_ has become an urgent task. To accurately assess the performance of CO_2_ separation by adsorption at the needed scale, adsorbents should be synthesized and fully characterized under the required operating conditions, and the proper design and simulation of the process should be implemented along with techno-economic and environmental assessments. Several works have examined pure CO_2_ single-component adsorption or binary mixtures of CO_2_ with nitrogen for different families of adsorbents, primarily addressing their CO_2_ adsorption capacity and selectivity; however, very limited data is available under other conditions and/or with impurities, mainly due to the intensive experimental (modeling) efforts required for the large number of adsorbents to be studied, posing a challenge for their assessment under the needed conditions. In this regard, molecular simulations can be employed in synergy with experiments, reliably generating missing adsorption properties of mixtures while providing understanding at the molecular level of the mechanism of the adsorption process.

This Account provides an outlook on strategies used for the rational design of materials for CO_2_ capture from different sources from the understanding of the adsorption mechanism at the molecular level. We illustrate with practical examples from our work and others’ work how molecular simulations can be reliably used to link the molecular knowledge of novel adsorbents for which limited data exist for CO_2_ capture adsorption processes. Molecular simulation results of different adsorbents, including MOFs, zeolites, and carbon-based and silica-based materials, are discussed, focusing on understanding the role of physical and chemical adsorption obtained from simulations and quantifying the impact of impurities in the performance of the materials. Furthermore, simulation results can be used for screening adsorbents from basic key performance indicators, such as cycling the working capacity, selectivity, and energy requirement, or for feeding detailed dynamic models to assess their performance in swing adsorption processes on the industrial scale, additionally including monetized performance indicators such as operating expenses, equipment sizes, and compression cost. Moreover, we highlight the role of molecular simulations in guiding strategies for improving the performance of these materials by functionalization with amines or creating hybrid solid materials. We show how integrating models at different scales provides a robust and reliable assessment of the performance of the adsorbent materials under the required industrial conditions, rationally guiding the search for best performers. Trends in additional computational resources that can be used, including machine learning, and perspectives on practical requirements for leveraging CO_2_ capture adsorption technologies on the needed scale are also discussed.

## Key References

- BalogunH. A.; BahamonD.; AlMenhaliS.; VegaL. F.; AlhajajA.Are we missing something when evaluating Adsorbents for CO_2_ Capture at the System Level?. Energy Environ. Sci.2021, 14, 6360–638010.1039/D1EE01677F.^1^ Integration of molecular simulation results with a process model of a pressure/vacuum swing adsorption process for CO_2_ capture in a commercial large-scale 550 MW coal plant using promising MOFs.
- ZhaoH.; BahamonD.; KhaleelM.; VegaL. F.Insights Into the Performance of Hybrid Graphene Oxide/MOFs for CO_2_ capture at Process Conditions by Molecular Simulations. Chem. Eng. J.2022, 449, 13788410.1016/j.cej.2022.137884.^2^ Molecular simulations are used to understand the CO_2_ adsorption mechanism on selected hybrid graphene oxide/MOFs to guide the experimental synthesis of optimized structures. The optimized model structures have comparable regeneration energy to industrially used amine scrubbing.
- BahamonD.; Díaz-MárquezA.; GamalloP.; VegaL. F.Energetic Evaluation of Swing Adsorption Processes for CO_2_ Capture in Selected MOFs and Zeolites: Effect of Impurities. Chem. Eng. J.2018, 342, 458–47310.1016/j.cej.2018.02.094.^3^ Assessment of the impact of impurities at the molecular level and process conditions on the performance of Mg-MOF-74, CuBTC, and zeolite 13X for CO_2_ capture, including working capacities, purities, recoveries, and exergetic requirements.

## 1 Introduction

In 2021, global CO_2_ emissions reached 36.3 gigatons,^4^ the highest ever annual level, with the power and industrial sectors being responsible for 40 and 23% of these emissions, respectively. The same year, the global economy increased by 5.9%, aligned with the 6% increase in emissions.^4^ Hence, one of today’s greatest challenges is providing sustainable energy to meet the demands for quality of life and economic growth without compromising the environment. Recognizing this urgency, some policies have been established, including the Paris Agreement, and strategies have been launched by several countries to reach net-zero emissions by 2050. This requires a radical technological transformation of the energy and industrial sectors with carbon capture, utilization, and storage identified^5^ as the key technologies to decarbonize them in the short to medium term.

CO_2_ capture is not new. CO_2_ has been traditionally separated from concentrated industrial sources for its applications in food and enhanced oil recovery, etc. For postcombustion CO_2_ capture, in which CO_2_ is removed from the flue gas, separation by absorption in aqueous amines is the standard technology used in industry. This is the first option for the strategies currently explored to reduce CO_2_ emissions to mitigate climate change since it is a mature technology and can be easily adapted to existing plants. Nevertheless, there are bottlenecks that need to be overcome, such as solvent degradation, losses due to evaporation, corrosion, and high energy requirements for regeneration.^6^ Thus, there is a need to find alternative solutions; active work is performed on searching for alternative solvents^7^ and other technologies to improve the efficiency of the capture processes and reducing their cost. In this Account, we focus on the advances performed in the last years searching for optimal porous adsorbents for CO_2_ capture, highlighting the role and benefits of using molecular simulations combined with process design to speed up this development.

## 2 Assessing Popular Studied Adsorbents for CO_2_ Capture

An ideal adsorbent for CO_2_ capture should display high gravimetric and volumetric adsorption capacities, high selectivity, minimal energy penalty for regeneration, long-term chemical, mechanical, and thermal stability under the operating conditions, and rapid gas diffusion, to mention some key factors.^8,9^ The separation from the flue gas is usually performed in a cyclic manner by swing adsorption (SA) processes, where regeneration is achieved by increasing the temperature (i.e., temperature swing adsorption, TSA) or lowering the pressure (i.e., pressure or vacuum swing adsorption, P/VSA). In addition, the selected adsorbent should be affordable and available on a large scale.

**Figure 1:**
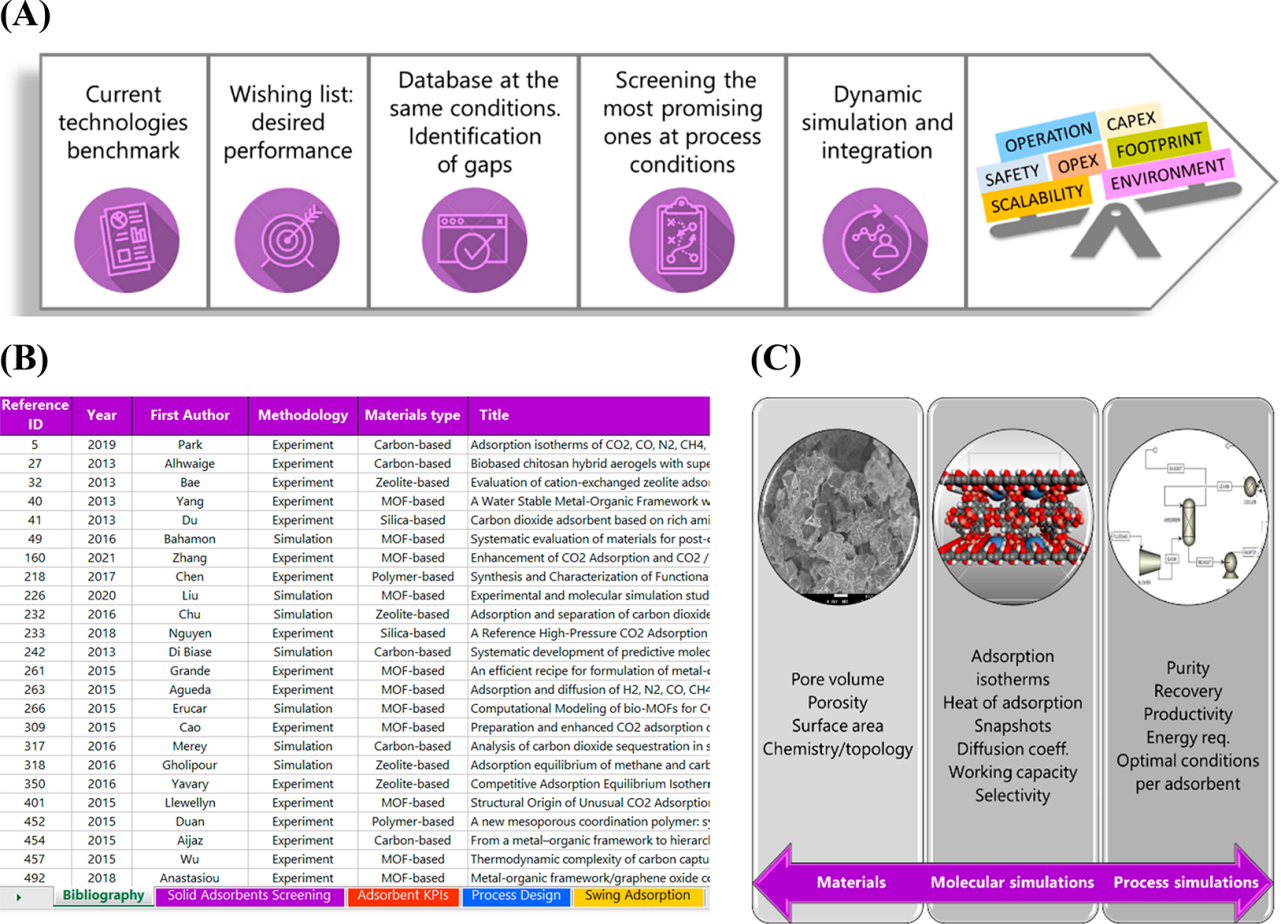
(A) Sketch of the approach followed to assess materials/processes for CO_2_ capture on a large scale. (B) Screenshot of the developed adsorbent database. (C) Materials’ properties needed as inputs for molecular modeling (left), properties obtained from molecular simulations (middle), and properties obtained from the detailed process modeling (right) to assess CO_2_ capture adsorption technologies.

As the search for efficient CO_2_ capture adsorbents is a hot research topic, several publications and patents appear every year, claiming excellent performance, while experiments (and simulations) are performed in some cases far from the industrial operating conditions. Commercially available and novel laboratory-scale synthesized adsorbents explored for CO_2_ capture include zeolites,^10^ activated carbons,^11−13^ zeolite-templated carbons (ZTC),^14−16^ metal–organic frameworks (MOFs),^8,17^ silica-based materials,^18,19^ metal oxides,^20^ and covalent organic frameworks (COFs),^21^ among others, as well as amine-functionalized versions of them, or hybrid materials, as will be discussed in the following sections. For a reliable comparison of them and in collaboration with the IAE Greenhouse-Gas R&D Program (IEAGHG)^22^ and the Abu Dhabi National Oil Company (ADNOC), we have established a procedure to screen available and novel systems for CO_2_ capture (solvents and adsorbents) under the same thermodynamic conditions and by standardizing the adsorption units for a fair comparison (see Figure 1(A)). A database of available publications and patents over the last 20 years was developed, including bibliographic references, adsorption data, key performance indicators (KPIs) such as heat of adsorption and selectivity, and their performance in SA processes under idealized regeneration conditions, when available. A screenshot of the database of adsorbents is shown in Figure 1(B).

As inferred from the collected data, most of the works deal with pure CO_2_ adsorption isotherms, or CO_2_/N_2_ separation under laboratory conditions, focusing on the CO_2_ capacity or binary selectivity. However, it has been demonstrated that some of the promising adsorbents do not perform well under realistic flue gas conditions, containing water^3,23^ and other impurities,^3^ or under the needed process conditions.^1^ The issue behind this lack of data is the large number of materials to be tested and the number of experiments needed to fully assess them. This is where molecular simulations come into play. These computational tools deliver macroscopic properties from the interactions of the molecules among them and with the adsorbent, which can be used in a reliable manner to generate data to feed the process design in the absence of experimental data. The most standard methods to study adsorbents are grand canonical Monte Carlo (GCMC), which provides adsorption isotherms, heats of adsorption, and the location of the molecules in the material, and molecular dynamics (MD), additionally providing transport properties such as diffusivity and viscosity inside the pores. Highlighted in Figure 1(C) are the type of properties collected from the materials, and calculated from molecular simulations and process design, for assessing the materials under the needed operating conditions.

To reliably use molecular simulations for predicting properties, the selected force fields for calculating the interaction between particles need to be validated with available experimental data (e.g., for CO_2_, using the adsorption isotherms at 273 K). Once validated, molecular simulations can be used to (1) understand the effect of chemistry and geometry on the capacity and selectivity of the adsorbent and the effect of impurities, allowing a first screening of them, (2) generate data, filling the gap in experimental data needed for the assessment under process conditions, and (3) feed detailed process modeling on an industrial scale. Our group uses this approach to reliably assess adsorbents for CO_2_ capture, as highlighted next.

## 3 Screening Adsorbents for CO_2_ Capture: Impact of Chemistry and Topology on Their Performance

It is well known that the adsorbent performance is directly related to inherent properties such as pore geometry and volume, surface area, and chemistry of the surface, all affecting the interactions with CO_2_ and accompanying molecules, as well as the available free volume for adsorption. Hence, understanding and being able to control these parameters would allow the fine-tuning of materials for desired applications. In this regard, MOFs stand as the paradigm of materials’ tunability. In 2011, Bae and Snurr^24^ published their pioneering work on evaluating 40 MOFs for CO_2_ capture, focusing on 4 tunable properties: pore size, open metal sites, polar functional groups, and the introduction of alkali metal cations. Their evaluation was based on five KPIs: CO_2_ uptake under adsorption conditions, working capacity, regenerability, selectivity, and the sorbent selection parameter, exclusively using available data of single-component isotherms and the ideal adsorbed solution theory for obtaining the selectivities. Although the procedure is not highly accurate, it provides a helpful framework for a first screening of the materials. Interestingly, they found correlations between the heat of adsorption of CO_2_ and the adsorbent evaluation criteria but no correlations with structural properties such as the pore size or surface area, highlighting the key role of the chemistry of the MOFs and the specific interactions of CO_2_ with the adsorbent for CO_2_ separation at low pressures.

**Figure 2:**
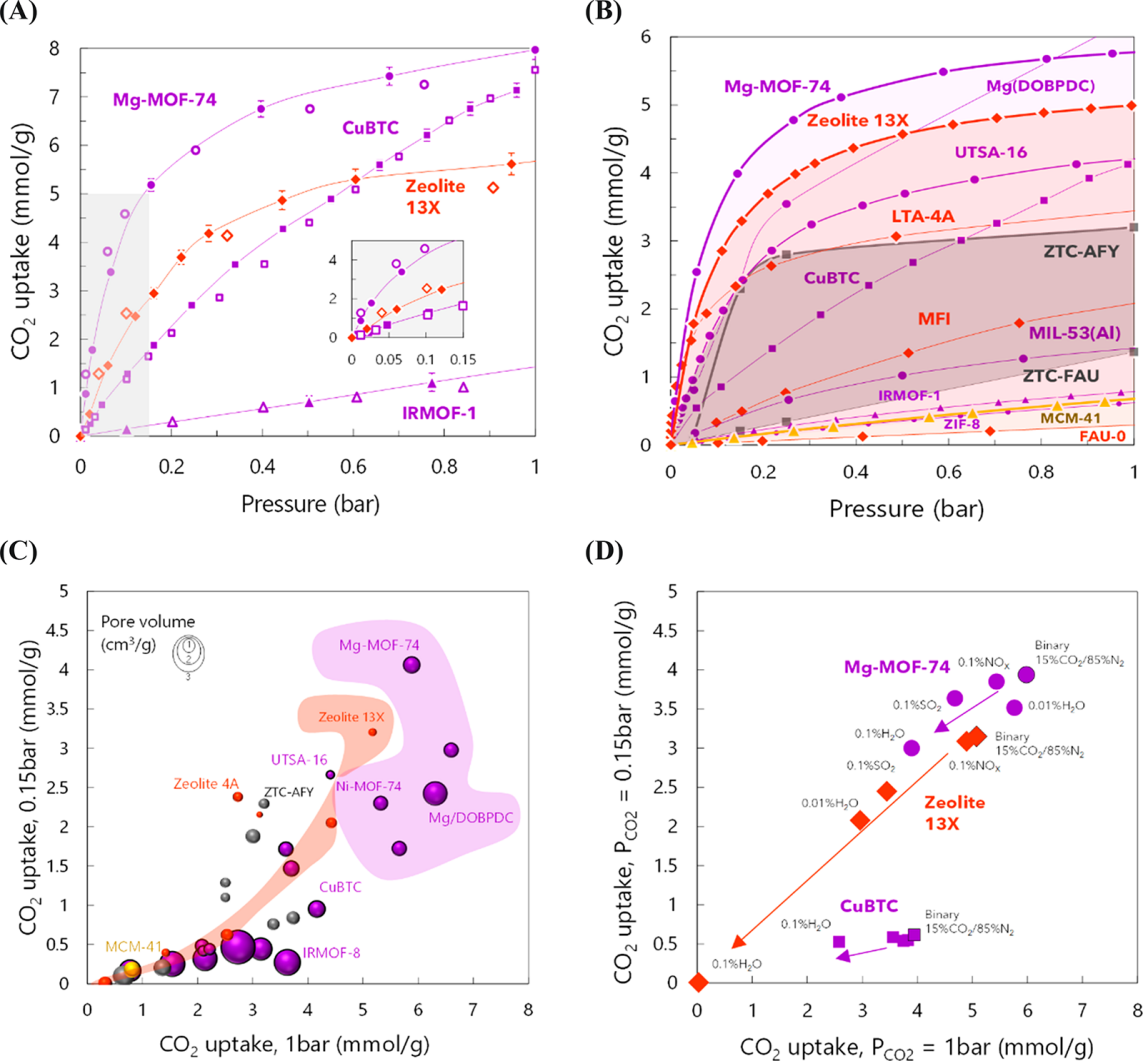
(A) Validation of pure CO_2_ adsorption isotherms with experimental data [*T* = 273 K].^1,3,8,12,25,26^ The inset in the figure is a zoomed-in view of the adsorption at low pressures. (B) Simulated adsorption isotherms of pure CO_2_ in selected materials [*T* = 313 K]. Highlighted areas represent other isotherms of same material type, not shown for the sake of clarity (color code: purple, red, gray, and yellow for MOFs, zeolites, carbon-based, and silica-based materials, respectively). (C) Comparison of simulated (pure) CO_2_ adsorption capacities under different total pressures [*T* = 313 K], where the size of the sphere represents the pore volume of the adsorbents. (D) Effect of impurities on the CO_2_ capacity of selected materials at two different partial pressures of CO_2_. In all cases, the ternary mixtures were simulated for 15% CO_2_, with nitrogen being present in surplus after the indicated concentration of traces. See the details in ref (3).

Five years later, we published a systematic computational study comparing several representative MOFs, zeolites, and other materials, focusing on the behavior of the adsorbents under industrial conditions from understanding the adsorbent performance at the molecular level and the impact of the chemistry/topology on it.^8^ After validation with available experimental data (see Figure 2(A) for 4 selected examples, with 5.8% being the highest observed average absolute deviation for CuBTC), adsorption isotherms, Henry’s constants, selectivities, and heats of adsorption were determined from GCMC simulations for 11 adsorbent families. In Figure 2(B), we present pure-component adsorption isotherms of the studied adsorbents in 2016,^8^ in combination with other promising materials from later studies^1,3,12,25,26^ at 313 K, the typical temperature of the flue gas for CO_2_ capture postcombustion. Calculated working capacities and breakthrough curves obtained from the molecular simulations were included for a TSA process, considering the effect of water traces as an impurity in a thorough comparison of the materials.^8^

We have used the data from these simulations to further evaluate the performance under process conditions, focusing on the influence of geometry and chemistry on the capacity and selectivity. In Figure 2(C), we showcase their capacities from a postcombustion stream when simulating pure CO_2_ (i.e., 1 bar, 313 K) and a binary mixture with similar compositions as the flue gas in a power plant (i.e., 15% CO_2_/85% N_2_, total pressure of 1 bar, 313 K). The purple highlighted region shows structures from the MOF-74 family, displaying the effect of using different metal centers or longer linkers; the highlighted region in red denotes the adsorption performance of faujasite-type zeolites with different Si/Al ratio. The size of the sphere represents the pore volume used to identify possible trends with respect to the shape/topology of the lattice. The top performer adsorbents found included Mg-MOF-74 and zeolite 13X, followed by UTSA-16, among others. As inferred from the figure, the pore volume is not critical for an *a priori* selection, as neither small nor large pores are required for this separation. In contrast, it is observed that the chemistry of the material, for the same topology, has a greater impact on their performance, showing similar saturation adsorption capacities but rather different uptakes at low pressures.

Mg-MOF-74 emerged as the most promising adsorbent to be used in SA processes, combining the high attraction of CO_2_ at low pressures (strong affinity with the metal centers) with the high capacity at high pressures (overall pore volume), closely followed by the benchmark zeolite 13X (slightly less attractive interactions, easier to desorb, and lower overall pore volume). Nevertheless, Mg-MOF-74 would require further detailed process modeling and experimental investigation on the right scale, with a caution of its cost in large-scale industrial implementation.

Furthermore, although carbon-based materials (represented by gray spheres) are not *per se* recommended adsorbents for this separation, due to weaker interactions of CO_2_ with the carbon surfaces, the intricate pore geometries of some ZTCs allow increasing their adsorption capacity and selectivity at low pressures.^15^ Promising ZTCs include ZTC-AFY and ZTC-IRR types,^27^ although with selectivity and other parameters below the recommended target values from the U.S. Department of Energy (DOE), thus limiting their use unless further modifications are performed.^27^

In a subsequent study, we further investigated three of the most studied (and top performer) adsorbents at that time—zeolite 13X, Mg-MOF-74, and CuBTC—explicitly evaluating the effect of impurities (O_2_, H_2_O, SO_2_, and NO_2_).^3^Figure 2(D) presents the influence of having traces of impurities in the performance of the materials for CO_2_ capture. In general, it is observed that the adsorption capacity decreases in the presence of impurities, marked with an arrow in the figure, as drastically pronounced for water in zeolite 13X. Although it is not shown in the figure, a hot spot^3^ was also found where, in some cases, small traces of unwanted gases actually helped to reduce the energy requirement per ton of CO_2_ captured (t_CO_2__) while increasing the recovery and purity of the collected stream (attributed to the introduction of competitive molecules in the flue gas, as inferred from the simulations), which is most evident for Mg-MOF-74. Nevertheless, there is an “optimal” inflection point where the competition for CO_2_-preferred adsorption sites produces a sharp exponential increase in the energy requirement.

**Figure 3:**
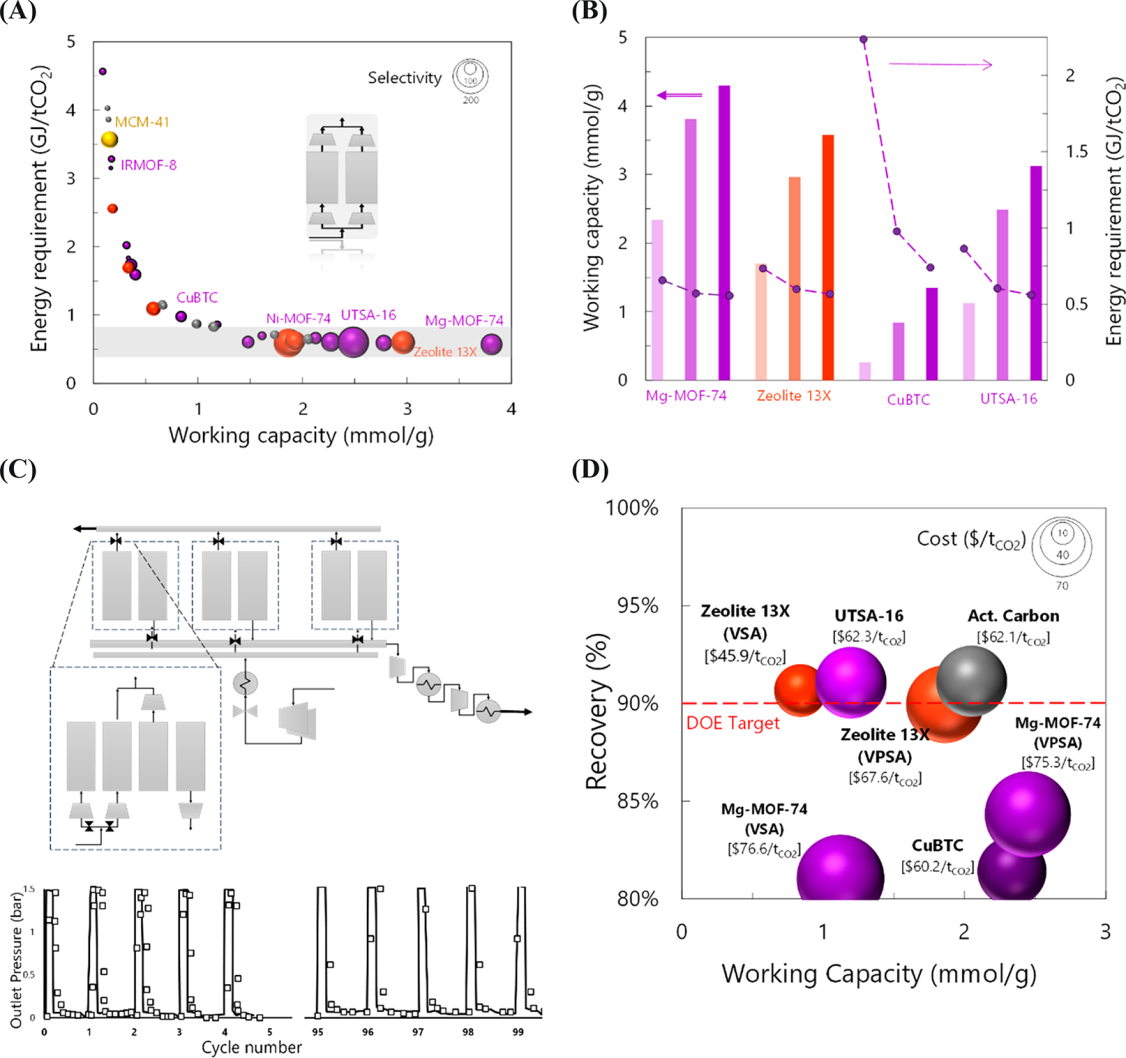
(A) Comparison of materials’ CO_2_ working capacity vs the energy required for desorption with data calculated from molecular simulations [Adsorption: 15% CO_2_/85% N_2_, *P* = 1 bar, *T* = 313 K; desorption: *P* = 0.2 bar, *T* = 393 K]. The size of the spheres represents the selectivity. Inset showing the shortcut scheme used to calculate the parameters. The gray region corresponds to comparable energy requirements for solvents.^28^ (B) Effect of inlet CO_2_ concentration in the obtained working capacity (left axis) and energy requirement (right axis) of selected materials. CO_2_ concentrations of 5, 15, and 25%, from lighter to darker color (N_2_ as surplus). (C) Top: Scheme used for the detailed model of the adsorption process at large scale. Bottom: Validation of the transient exit pressure profile over 100 cycles.^1^ (D) Comparison of working capacity vs CO_2_ recovery for detailed modeling. The size of the sphere corresponds to the total cost of the system, including CAPEX, compression train, utilities, materials replacement, etc. See details in ref (1).

Once the influence of the chemistry, topology of the materials, and presence of impurities on the CO_2_ capacity is assessed, simulation data can also be used to obtain the non-monetized KPIs of the process. Figure 3(A) depicts working capacities and energy requirements for the regeneration of the simulated adsorbent materials (in a similar manner as presented for materials’ adsorption capacities),^25,28^ emphasizing both adsorption and desorption conditions in the cycle (VTSA: adsorption 15% CO_2_/85% N_2_, 100 kPa, 313 K; desorption 0.2 kPa, 393 K) and taking advantage of the flexibility of molecular simulations to predict these properties in the absence of experimental data. Notice that in this case impurities are not explicitly considered to simplify the approach. The KPIs obtained show that zeolite 13X, Mg-MOF-74, and UTSA-16 remain at the top of the ranking, with conditions that can compete with those of most-mature absorption stripping with 30 wt % aqueous amines (region highlighted in gray). Notice that these results were obtained with a shortcut method,^25,26^ which indicates an upper bound of performance, with no temperature or concentration gradients in the adsorption bed during the adsorption/desorption cycle, allowing a simple description based only on equilibrium parameters, under the same conditions and bed size, regardless of the rate. Moreover, the effect of the inlet CO_2_ concentration in two KPIs is presented in Figure 3(B), showing that the energy requirement exponentially increases as the CO_2_ concentration is more diluted in the feed stream, especially for MOF CuBTC.

An analysis from the shortcut method allows an efficient comparison for initial screening, permitting a first ranking of promising adsorbents (Figure 3(A)). Once this is done, more rigorous process modeling is required for a detailed study of their performance under real industrial conditions, including the cost associated with them. Hence, the top performers from the shortcut method were further assessed,^1^ including MOFs Mg-MOF-74, CuBTC, and UTSA-16 as well as an activated carbon (AC) and zeolite 13X for comparative purposes and as a benchmark, respectively. Notice that despite the lower performance of CuBTC, it was included in the study for two main reasons: it showed good stability under wet conditions^3,8^ and it can be synthesized on a large scale.^29^ The developed multiscale modeling approach comprised molecular simulations, a process model of a P/VSA system, and techno-economic assessment including sensitivity analysis, mostly associated with uncertainties in the material cost, replacement rate, and compression. GCMC simulations provided equilibrium adsorption isotherms at different temperatures (based on CO_2_/N_2_ binary mixtures), heats of adsorption, and material structural features (e.g., density, pore volume), which were integrated into the detailed dynamic process to find the optimal operating conditions, design, and scheduling (e.g., number of columns) for each of the studied adsorbents. Minimization of the capture cost system was set as the objective function for each adsorbent, considering the operating conditions and process design.

The process model was first validated with experimental data, on a pilot scale^30^ (see the validation of the transient exit pressure profile over 100 cycles as an example in Figure 3(C)), and then used to assess the performance of an adsorption CO_2_ capture unit attached to a 550MW coal plant, aiming at achieving the 90/95% recovery/purity DOE target. Figure 3(D) depicts the recovery versus working capacity for the optimized conditions, where the size of the spheres represents the total capture cost (CAPEX and OPEX). Among the five studied materials, it was found that zeolite 13X can meet the target with an approximate capture cost of $45.9/t_CO_2__. UTSA-16 is highlighted as a very competitive adsorbent, achieving 90/92 recovery/purity and a capture cost of ca. $60/t_CO_2__. These results partially depend on the assumptions of the adsorbents’ cost: $10/kg was assumed for the MOFs versus the commercial cost of $0.5/kg of 13X and $0.3/kg of AC; for instance, assuming $1.25/kg for UTSA-16 can lead to a capture cost <45/t_CO_2__. Hence, attention needs to be paid to reducing the cost of UTSA-16, in addition to performing mechanical and stability tests to make it an industrially attractive option. Surprisingly, even though Mg-MOF-74 previously showed unbeatable equilibrium performance for any KPI with the shortcut method, the dynamic capture process showed that the DOE target is not met by this material, mostly due to a lower selectivity versus UTSA-16, but it might be efficient for achieving a 90/90 recovery/purity with a capture cost of ca. $70/t_CO_2__ (∼$55/t_CO_2__ for a material cost of $1.25/kg).

## 4 Strategies to Improve the Adsorbents’ Performance for CO_2_ Capture: Combining the Best of Top Performers

**Figure 4:**
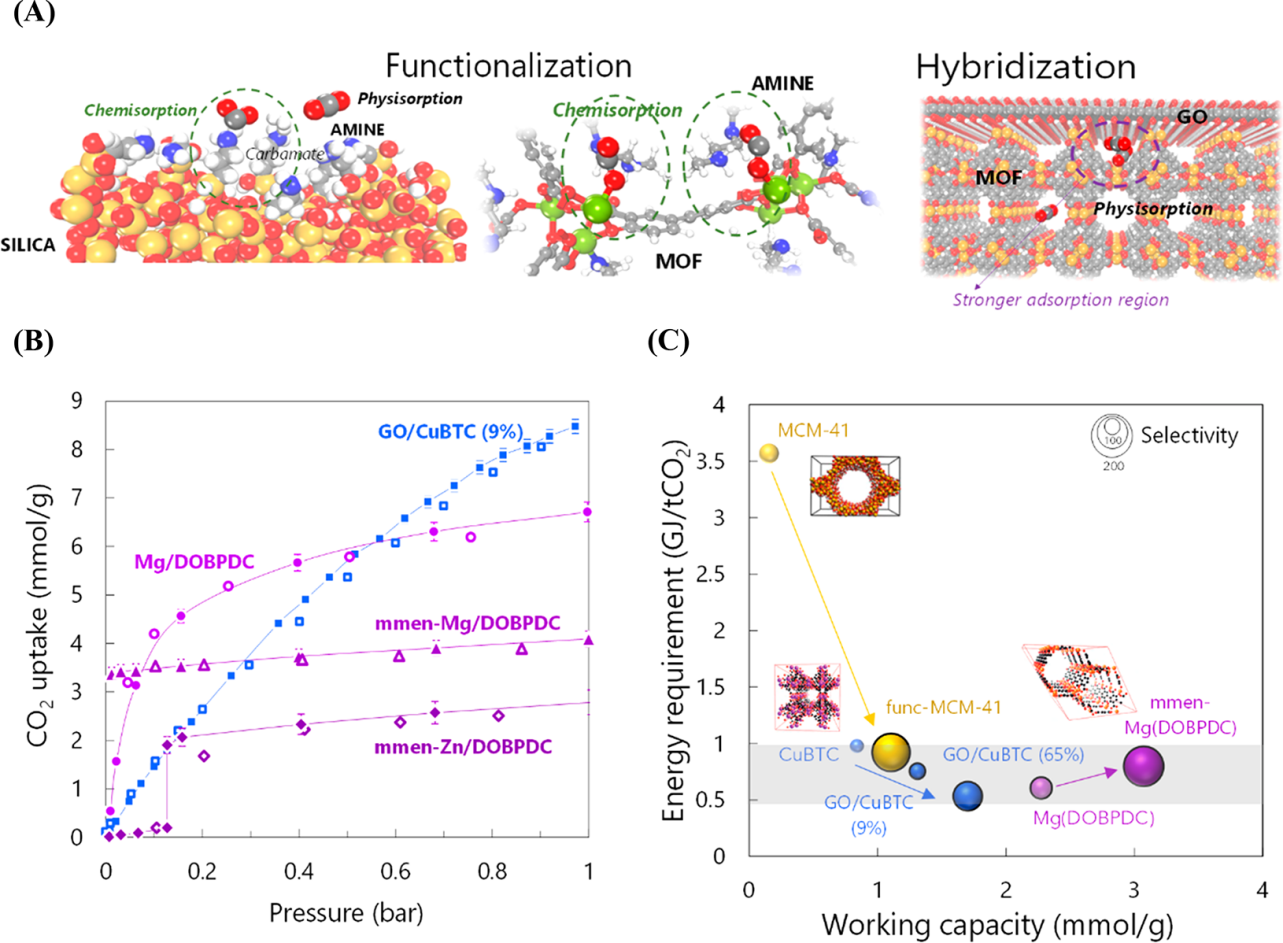
(A) Two strategies used for improving the performance of adsorbents for CO_2_ capture. Left: Functionalization with amine groups. Right: Hybridization of MOFs with GO. [Color code: yellow, gray, red, white, blue, green, and orange for Si, C, O, H, N, Mg, and Cu, respectively] (B) Validation of selected functionalized and hybridized materials versus experimental data. See details in refs (2) and (37). (C) Working capacity versus energy for the regeneration for CO_2_ capture, including selected functionalized and hybridized materials and the bare parents.^2,31,37^ The size of the spheres represents selectivity. Modified materials by functionalization or hybridization are shown with lighter spheres of the same color. The change in the performance from the bare materials to the hybridized or functionalized material is shown by an arrow.

As discussed, the chemistry and geometry of the adsorbents determine the interactions of CO_2_ and accompanying molecules with the material and hence the adsorption and kinetic processes. Knowing how materials behave at the molecular level and the underlying phenomenon of the adsorption allows devising strategies to improve their performance. From the different approaches taken in the literature, our group has been working on two of the most promising ones, synergistically combining experimental work with molecular simulations: functionalization with amine chains and hybridization of MOFs with graphene oxide (GO) (see Figure 4(A)).

Functionalization consists of adding functional groups to a support material either by wet impregnation or by anchoring them on the surface with covalent bonds. For CO_2_ capture, functionalization is used to increase the CO_2_ affinity, reduce the diffusion resistance, and enhance the selectivity, usually done with amine-based chains. This approach addresses two of the bottlenecks of the amines’ absorption technology: the loss by evaporation and the regeneration energy associated with heating the water used in the absorption column. Functionalization increases the interaction energy between CO_2_ molecules and the adsorbent surface, whereas it decreases the free available volume for adsorption; the overall efficiency is therefore a trade-off between these factors.

Several materials have been functionalized for CO_2_ capture, including silica-based materials,^31−34^ carbon nanotubes,^35^ and MOFs.^24,36−39^ Given the complexity of these materials, molecular simulations can greatly help in screening them under process conditions by isolating the role molecular interactions play in their performance and generating the needed data for their assessment.

Back in 2008, when functionalization for CO_2_ capture became a relevant topic and simulation works in this field were still scarce, we started a collaboration with Prof. Concepción Domingo at the Materials Science Institute of Barcelona to develop a modeling–experimental approach strategy for understanding at the molecular level the process of functionalization and its impact on improving the performance of silica-based materials for CO_2_ capture. Within the framework of S. Builes’ Ph.D. work, we proposed a simple yet accurate method to explicitly consider the chemisorbed CO_2_ on the amine groups^31^ that became a breakthrough in the field. Instead of just grafting amine chains, we assumed that the primary amines form a carbamate and a protonated base upon reaction with CO_2_. Hence, a predefined number of reacted amine chains were grafted to the solid materials. The number of these molecules, fixed beforehand, remained constant during the simulations. This assumption implies that CO_2_ reacts with the amine chains at very low pressures, while increasing the pressure has no further effect on the reaction, as proven afterward. The procedure was first applied to functionalized MCM-41, which showed excellent agreement with the experimental data. Several works followed, combining molecular simulations with experiments, exploring several types of adsorbents,^18,19,36^ putting emphasis on eco-friendly synthesis procedures while improving the CO_2_ capture performance, and obtaining good selectivity, fast adsorption/desorption rates, and chemical and thermal stability adequate for SA processes.

A similar procedure was recently applied by our group at the RICH Center to understand at the molecular level the role of functionalizing MOFs with diamines in increasing the performance of the counterpart MOFs for CO_2_ capture, first validating the procedure versus experimental data. Figure 4(B) depicts CO_2_ adsorption isotherms obtained from GCMC simulations, in excellent agreement with available experimental data for three selected functionalized MOFs. As an example of how functionalization affects the adsorption cycle performance, we depict in Figure 4(C) the working capacity versus energy for regeneration of adsorbents for CO_2_ capture, including functionalized and hybridized materials (same adsorption/desorption conditions as in Figure 3(A)). It is clearly observed that the functionalization (see mmem-Mg/DOBPDC) increases the working capacity and selectivity but at the expense of a higher energy requirement, pointing out the need to balance these outcomes for optimal capture in terms of performance and cost. Conversely, mesoporous MCM-41 silica shows massive improvement after being chemically functionalized.

Yet, functionalization is not the only approach used to improve the performance of adsorbents. In 2009, Petit and Bandosz^40,41^ proposed a way to generate hybrid materials of GO and MOFs, building on the strengths of both for gas separation. GO presents excellent mechanical and conductive properties, while MOFs are tunable materials with well-defined pore structure but are usually fragile, posing limitations to long-term use in adsorption packed beds.^42^ Thus, hybridization with GO (and other materials) is a promising approach to take advantage of their respective structural benefits and achieve the desired performance.

Some GO/MOFs have been synthesized and tested for CO_2_ capture on the laboratory scale.^40,43−49^ A common conclusion from these studies is that hybridizing the MOF with an appropriate amount of GO can enhance the adsorption capacity of CO_2_ at low pressures. However, due to the complexity of these hybrid materials, a clear understanding of the mechanism of adsorption and the role of the different parameters is difficult to infer solely by experimental work. Building on the experience of our team on combining molecular simulations with process design and the need to explore these types of materials under process conditions, we envisioned a strategy for understanding the mechanism of CO_2_ adsorption and separation on selected GO/MOFs, focusing on GO/CuBTC and GO/UTSA-16,^2^ for which some experimental data on CO_2_ adsorption were available. We first developed molecular models mimicking the experimental hybrid materials, using crystallographic information for both MOF and GO, and building a sandwich-like structure (see Figure 4(A)) inspired by the work of Petit and Bandosz.^40^ The force fields used for the adsorbents and gases were first validated, obtaining excellent agreement with experimental data (see Figure 4(B) for GO/CuBTC with 9% GO). A systematic study investigating the effect of different structural variables was performed, confirming that the interface between GO and MOFs produces strong interactions with CO_2_ (and small pores), which significantly enhances the uptake in the low-pressure range. Hypothetical GO/CuBTC with the highest GO content (i.e., 65 wt %) built with single layers of GO (no stacking) showed the best results in terms of selectivity (120 at 313 K), working capacity (1.68 mmol/g, TSA desorbing at 393 K), and energy requirement (0.53 GJ/t_CO_2__), comparable to amine scrubbing (see Figure 4(C)). However, we have not yet been able to synthesize it in the laboratory due to the difficulty of obtaining perfect “sandwich-like” structures with just one layer of GO at this very high concentration. Nevertheless, several other structures with lower GO content have been successfully synthesized, showing excellent agreement with simulation data, although their performance is not as good as the ideal hybrid with 65 wt % GO. Remarkably, as observed in Figure 4(C), hybridizing CuBTC with just 9% GO increases the working capacity and selectivity while decreasing the required energy per ton of captured CO_2_. Furthermore, even though CuBTC was not at the top of the ranking, its performance greatly improved if hybridized with GO, in a similar manner as happened with amine functionalization for other materials.

## 5 Summary and Outlook

As highlighted throughout this Account, CO_2_ capture by adsorption is a clear option to replace the mature absorption technology with aqueous amines, overcoming some of its limitations. However, despite the large number of publications exploring different adsorbent materials and their improvement by functionalization or hybridization, work is still needed to find the optimal adsorbents, assessing them under the industrial conditions in which they are going to be used before claiming their goodness just from limited measurements or calculations, far from these conditions.

We have shown how molecular simulations can help in the systematic search for adsorbent materials for CO_2_ capture. In addition to allowing an understanding of the performance of the adsorbents at the molecular level, identifying the role of chemistry/topology on the CO_2_ adsorption behavior, and how functionalization/hybridization can enhance the performance of these materials, they also allowed the calculation of KPIs needed for assessing the materials under process conditions to fill the gap of needed experimental data for process modeling. One of the insights gained from the study of functionalized materials is that, typically, parent materials showing a working capacity below 1 mmol/g can enhance their performance in terms of capacity, selectivity, and energy requirement while the functionalization of already-promising materials shows drawbacks, especially in energy terms. Additionally, we have quantified the advantages of hybridizing selected MOFs with GO by increasing the working capacity and lowering the regeneration energy.

The assessment of these adsorbents should first be performed using technical (non-monetized) KPIs such as the working capacity, selectivity, breakthrough curves, and energy requirement for regeneration, ranking them using, for instance, a shortcut method. However, one of the main learnings extracted from the detailed process modeling^1^ is that although non-monetized KPIs using a shortcut method greatly help in ranking the most promising adsorbents, the overall performance of the materials under the scaled plant conditions also depends on some additional parameters (e.g., the dynamics of the operating conditions, material cost, equipment sizing, etc.). A clear example is the case of Mg-MOF-74 versus zeolite 13X and UTSA-16, ranked as the top performer using solely technical KPIs but not when the detailed dynamic process model was implemented, although it is still a good candidate. Nevertheless, detailed process modeling has its own limitations for a thorough implementation as a relatively huge amount of data, not always available, is needed to perform it, and sensitivity analysis, such as the cost of the materials and other factors, should be performed for a reliable assessment. Hence, on the practical side, given the simplicity of the shortcut method, it is highly recommended to use it as a screening tool, as it is able to identify the top performers without the need for detailed modeling, unless detailed costs assessments are required.

Looking ahead, molecular simulations will continue to be an excellent complementary tool to experiments, but other computational techniques can be incorporated into the systematic search for adsorbents for CO_2_ capture, including guided machine learning^50^ to reliably cover the huge amount of materials and their available information. At the atomic and molecular levels, other techniques such as the implementation of reactive force fields^51^ can be used for the detailed analysis of the chemical reactions of CO_2_ with the adsorbents and other species. This will allow a detailed study of the degradation and recovery of the materials when they are continuously exposed to the flue gas. Furthermore, the multiscaling approach presented here, where molecular simulations are used as a tool filling the gap for process design, is not limited to processes for CO_2_ capture, and it can be used as a general procedure for the design of different chemical processes for which experimental data is missing.

Finally, the picture is not completed if thermal, chemical, and mechanical stability tests are not performed under real exposure conditions. Furthermore, the availability and low cost of the adsorbent materials should be considered to accelerate the implementation of this technology on the scale needed to mitigate climate change.
